# The Association between Relationship Satisfaction Concordance and Breast Cancer Survivors’ Physical and Psychosocial Well-Being

**DOI:** 10.3390/healthcare12020134

**Published:** 2024-01-08

**Authors:** Eric A. Vachon, Ellen Krueger, David A. Haggstrom, Victoria L. Champion

**Affiliations:** 1 School of Nursing, Indiana University, Indianapolis, IN 46202, USA; 2 Center for Health Services Research, Regenstrief Institute, Indianapolis, IN 46202, USA; 3 Department of Psychology, Indiana University-Purdue University Indianapolis, Indianapolis, IN 46202, USA; 4 School of Medicine, Indiana University, Indianapolis, IN 46202, USA

**Keywords:** cancer survivorship, relationship satisfaction, concordance, psychosocial

## Abstract

The purpose of this article is to examine the association of relationship satisfaction concordance between breast cancer survivors (BCSs) and their partners with matched controls on physical and psychosocial outcomes. Dyads of BCSs, age-matched controls, and partners were recruited as part of a larger, cross-sectional QOL survey study. Relationship concordance was measured by the ENRICH marital satisfaction score, with each dyad’s score equaling the absolute value of the difference in satisfaction between survivor/control and their partner (lower score = greater concordance). Dependent variables for survivors/controls were social constraint, physical function, depression, fatigue, attention function, and sleep disturbance. Relationship satisfaction and concordance were used as the primary independent variables, while controlling for dyad category, race, education, income, and age within multiple linear regression models. The sample consisted of 387 dyads (220 BCSs, 167 controls). Relationship satisfaction concordance ranged from 0 to 53.4 (mean = 10.2). The BCS dyads had significantly worse concordance (11.1) than the controls (9.1) (*p* = 0.050). Within the multiple regression models, lower concordance was significantly associated with increased social constraint (*p* = 0.029), increased depression (*p* = 0.038), and increased fatigue (*p* = 0.006). Poor relationship satisfaction and concordance were significantly associated with poor physical and psychosocial outcomes. The maintenance of relationships should remain a focus through difficulties of cancer and into survivorship for survivors, partners, and providers.

## 1. Introduction

Breast cancer (BC) is the most prevalent cancer diagnosis among women in the United States, with an estimated 3.8 million women living with a history of breast cancer [1,2]. Increased screening rates and improved treatments have resulted in a 90% five-year survival rate for women with breast cancer [1,3]. While breast cancer survival rates have continued to increase, breast cancer survivors (BCSs) are at risk of a myriad of short-term and long-term side effects. In the short term, BCSs may experience physical symptoms, including fatigue and limitations in functioning. In the long-term, BCSs report long-term physical, psychological, social, and spiritual difficulties [4]. In addition to the impact of BC on the physical and mental health of survivors, BC has the potential to impact social aspects for BCSs as well, particularly their partners and the overall quality and satisfaction of their relationships [5]. While partners may serve as a primary pillar of social support for BCSs, BC may impact the relationship satisfaction between BCSs and their partners, in turn resulting in a diminished sense of social support, leading to worsening physical and psychosocial outcomes and well-being [6,7].

Breast cancer survivors’ relationship satisfaction has been associated with a variety of psychosocial and physical outcomes, including less fatigue [8], decreased general and cancer-specific stress [8,9], lower depressive symptoms [9,10,11], better overall quality of life [12], increased resilience [11], and reduced fear of cancer recurrence [13]. In fact, BCSs in distressing relationships reported slower declines in cancer-specific distress and symptoms up to five years post-diagnosis compared to those in non-distressed relationships [9]. Many communication and coping strategies have also been associated with relationship satisfaction. For example, while cancer-related avoidance [14,15], negative dyadic coping [16], buffering [17], holding back feelings, and criticizing one’s partner [18] have been associated with decreased relationship satisfaction in cancer patients, more active and common dyadic coping [11,16] have been related to increased relationship satisfaction.

Little is known about whether survivors and their partners agree on their perceived relationship satisfaction and the relationship between satisfaction concordance and long-term outcomes. While one study with recently diagnosed cancer patients, across various cancer sites, and their caregivers (54% partners) found that relationship quality concordance was generally low, this varied across dyads and was not related to patient outcomes within one year post-diagnosis [19]. While relationship satisfaction, from the perspective of the survivor or the partner, may be high, that does not mean that concordance within the dyad relating to relationship satisfaction will be high. Poor concordance may negatively impact areas of survivors’ and partners’ lives. Thus, further research is needed to understand the associations between the concordance of relationship satisfaction and long-term breast cancer survivors’ psychosocial and physical outcomes.

The purpose of the present study was to (1) determine the relationship satisfaction concordance between BCSs and age-matched controls with their partners, respectively, based on each individual’s relationship satisfaction score; (2) explore the differences in relationship satisfaction and concordance, as well as psychosocial and physical outcomes between BCSs and age-matched controls; and (3) examine the impact of individual relationship satisfaction and dyad relationship concordance on different physical and psychosocial outcomes. We hypothesized that we would observe (1) lower relationship concordance in the BCS dyads than age-matched control dyads, and (2) that overall, worse relationship satisfaction and relationship concordance would be associated with poor physical and psychosocial outcomes. We expect that this work will lead to both clinicians and researchers working with BCSs better understanding the importance of social support structures and relationships with their partners. As a result, this will set up future work to both include relationship-focused variables and involve partners within studies and intervention development.

## 2. Methods

Data utilized for this secondary analysis were collected as part of a large, cross-sectional study focused on evaluating the effect of breast cancer on overall quality of life (QOL) and other physical and psychosocial metrics among women within two different groups: younger BCSs (≤45 years old at diagnosis) and age-matched controls [4]. Women in partnered relationships (married or long-term commitment), as well as their partners, were asked to complete additional measures to assess social indices. Analysis for the current study focused on younger BCSs and age-matched controls and their partners.

### 2.1. Sample and Recruitment

Women were identified using the Eastern Oncology Cooperative Group—American College of Radiology Imaging Network (ECOG-ACRIN) statistical center database. These women were a part of the ECOG-ACRIN database after previously being involved in one of the center’s clinical trials across 97 different sites. Eligibility criteria for the parent study were women who (1) were ≤45 years at initial cancer diagnosis at stages I–IIIa; (2) were 3–8 years post initial treatment at time of enrollment; (3) had no BC recurrence; and (4) had been treated with an adjuvant chemotherapy regimen, including Adriamycin, Paclitaxel, and Cyclophosphamide in order to reduce treatment-related variance.

After eligible BCSs were identified through the ECOG-ACRIN database and institutional review board (IRB) approval obtained (1009001681R007), the list of eligible BCSs was provided to each study site. Oncologists and clinical staff at each participating site briefly discussed the study with eligible BCSs and asked permission for the research team to contact them to discuss the study further. If survivors agreed, study sites provided survivors’ contact information to the research team, and survivors received a study brochure prior to contact with the team. After receiving the study brochures, research assistants followed up with eligible BCSs to obtain verbal consent to join the study. After verbal consent, BCSs were mailed a hard-copy consent form, as well as the study questionnaire.

In addition to asking for verbal consent, BCSs were asked if their partner might be willing to participate in the study; with partner agreement, a consent form and partner questionnaire was also mailed. Survivors were also asked to provide names and contact information for three women within five years of their current age and who did not have a breast cancer diagnosis, in order to be used as an age-matched control. The healthy controls allowed for comparison to better understand the impact of cancer on the various QOL domains being examined. Not all survivors provided three women for potential age-matched controls. After compiling a list of potential age-matched controls, accrual began, following the same procedure used for survivors. The full recruitment and procedure details can be found in the primary study paper [4].

For the parent study, a total of 744 eligible younger BCSs were contacted, with 86% verbally consenting and 67% (n = 505) completing the study questionnaire. For this secondary data analysis, the sample was limited to those with partners participating, resulting in 387 dyads (220 BCSs and 167 controls), which was ~40% for BCSs and ~48% for controls, respectively. Although both male and female partners were eligible, all partners that consented to participate were male.

### 2.2. Measures

The instruments completed by BCSs, age-matched controls, and each groups’ respective partners included questions related to sociodemographic factors (age, race, education, marital status, and household income), among other variables. Relationship satisfaction and relationship concordance were used as the primary independent variables, which are two distinct variables—concordance being derived from satisfaction scores (see calculation explanation below). The dependent variables were survivor/controls’ partner social constraint, physical function, depression, fatigue, attention function (performing daily tasks), and sleep disturbance.

Relationship Satisfaction And Relationship Concordance. The primary independent variables for this secondary analysis were relationship satisfaction and relationship concordance. Relationship satisfaction was measured using the ENRICH Material Satisfaction (EMS) Scale, which is a 15-question tool evaluating general marital satisfaction, relationship issues, communication, and happiness using a five-point Likert scale [20]. For the included sample, the EMS had an internal consistency of α = 0.91 for cases and α = 0.91 for controls. Relationship concordance was an aggregate measure of each dyad’s combined martial satisfaction score. Each dyad’s relationship concordance scale equaled the absolute value of the difference in satisfaction between survivor/control and their partner (i.e., BCS EMS score − partner score = dyad concordance). Therefore, a lower score reflected greater relationship concordance within pairs. Relationship satisfaction is the individual score, while relationship concordance is a single score for the dyad.

Partner Social Constraint. Partner social constraint was measured using the Social Constraint—Partner/Spouse scale developed by Lepore et al. [21] The instrument includes 14 items on a four-point Likert scale, which examine communication and response to stressful events with spouse/partner. It should be noted that although the questions of the scale are the same, the prompts used for BCSs asked specifically about situations related to breast cancer, while the questionnaire for controls asked about any situation or problem, in general. A higher social constraint scale score indicates greater partner/spouse constraint. For the included BCSs and controls, social constraint scale had an internal consistency of α = 0.89 for cases and α = 0.93 for controls.

Physical Function. Physical functioning was measured using the 36-item Short-Form Health Survey (SF-36) [22]. Ten items from SF-36 were used within the parent study (items 3–12 of original SF-36). These ten items utilized a three-point Likert scale, with a higher score indicating better physical functioning. The rationale for reducing the SF-36 to 10 items was in order to reduce participant burden, while still maintaining reliability and validity of the measure of physical functioning. The ten-item scale had an internal consistency of α = 0.88 for cases and α = 0.86 for controls.

Depression. Depression was measured using the Center for Epidemiologic Studies—Depression (CES-D) Scale [23], which was designed to measure depressive symptomology. The CES-D includes 20 items using a four-point Likert scale, with a higher score indicating increased presence of depressive symptomology. For the included sample, the CES-D had an internal consistency of α = 0.89 for cases and α = 0.90 for controls.

Fatigue. Fatigue was measured using the Functional Assessment of Cancer Therapy Fatigue (FACT-F) Subscale [24]. The FACT-F consists of 13 items using a five-point Likert scale, with a higher score indicating less fatigue. For the included sample, the FACT-F had an internal consistency of α = 0.95 for cases and α = 0.94 for controls.

Attention Function. An individual’s perceived cognitive function and ability to perform daily tasks were measured using the Attentional Function Index (AFI) [25]. The AFI includes 16 items using a 0–10 scale, with a higher score indicating greater perceived cognitive functioning. For the included sample, the CES-D had an internal consistency of α = 0.95 for cases and α = 0.94 for controls.

Sleep Disturbance. Sleep quality and disturbance was measured using the Pittsburgh Sleep Quality Index (PSQI) [26]. The PSQI includes 19 items using a four-point Likert scale, with higher score indicating poorer quality of sleep. Per Buys et al. [26], internal consistency should be measured per 7 component scores, rather than 19 item scores. For the included sample, the PSQI had an internal consistency of α = 0.75 for cases and α = 0.73 for controls.

### 2.3. Statistical Analysis

Frequencies and measures of central tendency were calculated for sociodemographic variables. Descriptive statistics were calculated to characterize sociodemographics, individual relationship satisfaction, relationship concordance, partner social constraint, physical function, depression, fatigue, attention function (performing daily tasks), and sleep disturbance. Two sample *t*-tests and Pearson chi-square tests were used to compare differences in sociodemographics between groups; no differences were found. Two sample *t*-tests compared individual relationship satisfaction, relationship concordance, partner social constraint, physical function, depression, fatigue, attention function (performing daily tasks), and sleep disturbance between BCSs and age-matched controls. For the primary analyses, six separate multiple linear regressions were run for each of the dependent variables (partner social constraint, physical function, depression, fatigue, attention function (performing daily tasks), and sleep disturbance). Individual relationship satisfaction and relationship concordance were used as the primary independent variables, while controlling for dyad category (BCS and control), age, race, education, income, and marital status within multiple linear regression models. Data were analyzed using STATA 17.0. While the actor–partner interdependence model [27] is frequently used for dyadic data sets such as this, this analytic approach is not appropriate given that the dependent variables are specific to survivors and controls only and not their partners.

## 3. Results

A total of 387 dyads were included in this secondary analysis, with 220 BCS dyads and 167 age-matched control dyads, for a total of 774 individuals. Table 1 includes the relevant sociodemographic variables for each of the dyad groups. The average age of BCSs was 45 years old, with their partners being 48 years old on average, while the average age of controls was 46 years old, with their partners being 47 years old on average. The sample was primarily white, well educated, married, and with a household income of between USD 50,000 and USD 100,000. Although everyone in the sample was a part of a dyad, a small portion of the sample did list their official marital status as divorced, widowed, or single (19, 5%), while still including their current partner within the study. For BCSs, the majority were stage II breast cancer at the time of diagnosis (65%), and they were about 6 years on average out from diagnosis at the time of study enrollment.

### 3.1. Relationship Satisfaction and Physical and Psychosocial Outcomes

Bivariate analyses for relationship satisfaction, relationship concordance, and physical and psychosocial outcomes for the total sample and BCSs and controls are reported in Table 2. The sample had a mean relationship satisfaction score of 52.4 (range: 8–88), with no difference between BCSs (52.0) and controls (53.0) (*p* = 0.497). For relationship concordance within dyads, BCS dyads (11.1) had significantly worse concordance than control dyads (9.1) (*p* = 0.050).

When examining the various physical and psychosocial outcomes, the individual scores for the BCSs and controls were used, as opposed to a combination of within-dyad scores. For partner social constraint, controls had significantly worse partner constraint than BCSs (*p* < 0.001). There was no difference in terms of physical functioning between BCSs and controls (*p* < 0.172). Survivors had significantly higher depressive symptomology than both controls (*p* = 0.010). There was no difference between BCSs and controls in terms of fatigue (*p* = 0.079). When looking at attention function, BCSs had lower perceived cognitive function on daily tasks than controls (*p* = 0.001). Finally, BCSs rated their sleep quality significantly worse than controls (*p* = 0.002).

### 3.2. Multivariate Analyses of Physical and Psychosocial Outcomes

Table 3 reports the multiple linear regression results for each of the six outcome variables, with relationship satisfaction and relationship concordance as the primary independent variables for each of the outcomes for a total of six regression models. After controlling for dyad category, age, race, education, and income level, lower individual relationship satisfaction was associated with poor partner social constraint (*p* < 0.001), worse physical functioning (*p* = 0.020), higher depressive symptomology (*p* < 0.001), increased fatigue (*p* < 0.001), lower perceived cognitive function on daily tasks (attention function) (*p* < 0.001), and poorer perceived sleep quality (*p* = 0.025), while worse dyad relationship concordance was associated with poor partner social constraint (*p* = 0.029), higher depressive symptomology (*p* = 0.038), and increased fatigue (*p* = 0.006). Relationship concordance was not significantly associated with physical functioning, attention function, or sleep disturbance.

Looking at the covariates within the models, BCSs had lower partner constraint (*p* < 0.001), increased depressive symptomology (*p* = 0.029), worse attention function (*p* < 0.005), and poorer sleep quality (*p* < 0.003) than controls. We found that as age increased, physical functioning decreased (*p* = 0.050). Finally, we found that non-white participants had worse partner social constraint than white participants (*p* = 0.049). As discussed in the analysis section, education and income levels were also included as covariates. However, neither of these were significant within the multivariable regression models.

## 4. Discussion

The purpose of this secondary analysis was to (1) determine the relationship satisfaction concordance between BCSs and age-matched controls with their partners, respectively; (2) explore the differences in relationship satisfaction and concordance, as well as psychosocial and physical outcomes between BCSs and age-matched controls; and (3) examine the impact of individual relationship satisfaction and dyad relationship concordance on different physical and psychosocial outcomes. While some prior research has been carried out examining marital/relationship satisfaction among breast cancer survivors, this is the first study to our knowledge that examined relationship concordance among BCSs and their partners. Additionally, our work included controls and their partners that were matched based on age to the BCSs. We also examined the impact of relationship concordance on various physical and psychosocial outcomes, which is a unique contribution to the literature. Only individual BCS relationship satisfaction has been associated with certain psychosocial outcomes within the literature, but these studies did not account for the partners’ perspectives [8,9,10,11,12,13,14,15,16,17,18].

As hypothesized, we found that BCS dyads had worse relationship concordance than age-matched control dyads. However, we did not find a significant difference in individual relationship satisfaction between BCSs and controls, indicating that once the perspective of the partner of a BCS is factored in, we see less agreement on the quality and satisfaction of their relationship than in controls pairs. This may not be surprising given that cancer has been overwhelmingly found to be associated with a number of mental health issues, particularly depression and anxiety, as well as poor physical outcomes, due to the nature of the disease [28,29,30,31,32,33]. Poor relationship satisfaction and concordance has the potential to exacerbate these physical and psychosocial outcomes, as was found in our work. It is noteworthy that we do not see a difference in individual ratings between BCSs and controls, but when factoring in the partners’ perspective, we do see a significant difference, given that this comparison has not been performed in the current literature.

The diagnosis of cancer and its treatment is a significant stressor for breast cancer survivors and their partners and can impact their relationship. Optimal communication is necessary to support a healthy relationship, quality of life, and continued success in managing cancer and its treatment [8,10,11]. When communication is poor between a couple dealing with cancer, this may lead to a number of relationship concerns, including communication, resentment, poor coping, holding back of feelings, and avoidance [34,35,36,37]. In our study, looking at the age-matched controls, these concerns are evident, with BCSs having poorer relationship satisfaction concordance, greater depressive symptoms, poor attention function, and poor sleep quality.

One concept that should be noted is partner social constraint. We found that the age-matched controls had worse partner constraint than BCS. This is likely due to an adjustment in the instrument for cancer survivors vs. controls. The cancer survivors’ partner constraint tool specifically asked about breast cancer as the focus of constraint within the relationship, while the controls’ tool asked about “problem or problems” in general. The specificity of the survivors’ tool to breast cancer likely limited the reported constraint, particularly with this sample not actively going through cancer treatment. Although the instruments varied slightly [21], we still found it useful to compare BCSs and controls given the focus of this work being relationship satisfaction and concordance.

For our primary research question, as hypothesized, we found that both lower relationship satisfaction and lower dyad relationship concordance were associated with worse partner social constraint, higher depressive symptomology, and increased fatigue. Individual relationship satisfaction was also significantly associated with worse physical functioning, worse attention function, and poorer sleep quality, while relationship concordance was not. Interestingly, the correlation between relationship satisfaction and relationship concordance was somewhat weak at −0.34. Given this low correlation, it is not true that BCSs or controls who rated their relationship satisfaction as high were certain to have more agreeable concordance with their partner.

It is important to examine both relationship satisfaction individually for survivors, as well as relationship satisfaction as a whole within survivor/partner dyads. In spite of this weak correlation, we observed similar results between relationship satisfaction and relationship concordance. We can confidently say that both poor individual relationship satisfaction and dyad concordance are strongly associated with a number of poor physical and psychosocial outcomes. While the psychology literature has continued to show that poor relationship satisfaction among the general population is associated with worse psychosocial outcomes, little has been done in this area for BCSs. This is the first study, to our knowledge, that has examined this phenomenon at another level by evaluating relationship concordance among BCSs and their partners, as well as comparing with age-matched controls.

## 5. Limitations

A number of limitations should be acknowledged within the parent study and this secondary analysis. First, while our sample was quite large in terms of the number of dyads, the sample was not diverse, with 95% being white. The sample is more highly educated and has a higher household income than the average population, and therefore, this reduces our generalizability. Specific to this secondary work, only 43% of the parent study sample had an included partner and were included within this secondary analysis. All partners within this sample were male. Given the increased percentage of same-sex couples in the US, there should be caution in generalizing these results to BCSs and same-sex partners. Looking at the data collection methods, the parent study was a cross-sectional design. While this secondary analysis did find several significant associations between relationship satisfaction/concordance and the included physical and psychosocial outcomes, we are unable to infer a cause and effect/directional relationship between the variables.

## 6. Conclusions and Implications for Cancer Survivors

In conclusion, our study is one of the few to examine the dynamic between breast cancer relationship satisfaction and physical and psychosocial outcomes. It is the first, to our knowledge, to examine the impact of relationship concordance between BCSs and their partner and these outcomes. We found that BCSs and their partners have lower relationship satisfaction concordance than age-matched control dyads. We found that both lower individual relationship satisfaction and worse dyad relationship concordance was significantly associated with a number of poor physical and psychosocial outcomes, regardless of dyad category. For BCSs and their partners, although it may be difficult at any point during the cancer care trajectory, this work points to the critical importance of both members of the couple focusing on strengthening the relationship. Difficulties among couples can have devastating effects for your physical and emotional health. For clinicians, making sure that based upon the preferences of the BCS, partners are involved in discussions, treatment, and overall care is vital to the short-term and long-term health of patients. For researchers, future work focused on physical and psychosocial outcomes should at the very least include relationship/marital variables, but more importantly, should also involve the BCS’s partner, whenever possible.

## Figures and Tables

**Table 1:** Comparison of characteristics between BCSs and controls.

Variable	Total Sample (N = 387)	BC Survivors (n = 220)	Controls (n = 167)	*p*-Value
Age (years), mean (SD)	45.5 (6.0)	45.3 (4.7)	45.8 (7.4)	0.491
Race, n (%) White Non-White	362 (93.5) 25 (6.5)	206 (93.6) 14 (6.4)	156 (93.4) 11 (6.6)	0.930
Education, n (%) High school or less Some college or trade school Associate or Bachelor’s degree Some or complete graduate school	59 (15.3) 92 (23.7) 147 (38.0) 89 (23.0)	39 (17.7) 57 (25.9) 71 (32.3) 53 (24.1)	20 (12.0) 35 (21.0) 76 (45.5) 36 (21.6)	0.063
Current Marital Status, n (%) Married (or long-term commitment) Divorced Widowed Single	368 (95.1) 8 (2.1) 2 (0.5) 9 (2.3)	208 (94.6) 6 (2.7) 1 (0.5) 5 (2.3)	160 (95.8) 2 (1.2) 1 (0.6) 4 (2.4)	0.769
Income, n (%) USD 0–USD 50,000 USD 50,001–USD 100,000 >USD 100,000 Don’t know or did not answer	59 (15.3) 187 (48.3) 138 (35.7) 3 (0.8)	30 (13.6) 107 (48.6) 82 (37.3) 1 (0.5)	29 (17.4) 80 (47.9) 56 (33.5) 2 (1.2)	0.588

SD, Standard Deviation.

**Table 2:** Comparison of relationship satisfaction and physical and psychosocial outcomes between BCSs and controls.

Variable, Mean (SD)	Total Sample (N = 387)	BC Survivors (n = 220)	Controls (n = 167)	*p*-Value
Relationship Satisfaction (Individual)	52.4 (13.5)	52.0 (14.1)	53.0 (12.7)	0.497
Relationship Concordance	10.2 (9.9)	11.1 (10.8)	9.1 (10.0)	**0.050**
Partner Social Constraint	23.2 (8.9)	20.3 (6.3)	26.2 (7.9)	**<0.001**
Physical Function	86.2 (17.7)	85.1 (18.6)	87.6 (16.4)	0.172
Depression	9.2 (8.6)	10.2 (8.9)	7.9 (8.1)	**0.010**
Fatigue	39.9 (10.5)	39.1 (10.9)	41.0 (9.6)	0.079
Attention Function	6.8 (1.7)	6.5 (1.8)	7.1 (1.6)	**0.001**
Sleep Disturbance	6.0 (3.5)	6.5 (3.6)	5.4 (3.3)	**0.002**

Bolded *p*-values indicate a significant coefficient at the 0.05 level. SD, Standard Deviation.

**Table 3:** Multiple linear regression results on physical and psychosocial outcomes (N = 387).

Outcomes	Social Constraint		Physical Function		Depression		Fatigue		Attention Function		Sleep Disturbance	
Independent Variables	Coef. (SE)	*p*-Value	Coef. (SE)	*p*-Value	Coef. (SE)	*p*-Value	Coef. (SE)	*p*-Value	Coef. (SE)	*p*-Value	Coef. (SE)	*p*-Value
Relationship Satisfaction	−0.239 (0.03)	**<0.001**	0.163 (0.06)	**0.020**	−0.244 (0.03)	**<0.001**	0.185 (0.04)	**<0.001**	0.039 (0.01)	**<0.001**	−0.033 (0.01)	**0.025**
Relationship Concordance	0.090 (0.04)	**0.029**	−0.088 (−1.2)	0.225	0.084 (0.04)	**0.038**	−0.144 (0.05)	**0.006**	−0.015 (0.01)	0.076	0.026 (0.02)	0.166
Dyad Category Control BCS	–– −5.62 (0.79)	**<0.001**	–– −2.05 (1.7)	0.238	–– 1.73 (0.79)	**0.029**	–– −1.17 (1.0)	0.258	–– −0.479 (0.17)	**0.005**	–– 1.09 (0.36)	**0.003**
Age	−0.071 (0.06)	0.283	−0.282 (0.14)	**0.050**	−0.030 (0.06)	0.644	0.022 (0.09)	0.791	0.015 (0.01)	0.272	0.028 (0.03)	0.352
Race Non-White White	–– −3.17 (1.6)	**0.049**	–– −2.47 (3.5)	0.484	–– −1.47 (1.6)	0.361	–– 2.82 (2.1)	0.179	–– 0.071 (0.34)	–– 0.828	–– −0.449 (0.73)	0.541

Each individual linear regression model also controlled for education, marital status, and income level, but these were non-significant in the models. Bolded *p*-values indicate a significant coefficient at the 0.05 level. Coef., Coefficient; SE, Standard Error.

## Data Availability

The data that support the findings of this study are available from the corresponding author upon reasonable request.
